# Twenty Years of Experience on Stem Cell Transplantation in Iran

**DOI:** 10.5812/ircmj.1915

**Published:** 2013-02-05

**Authors:** Ardeshir Ghavamzadeh, Kamran Alimoghaddam, Fatemeh Ghaffari, Roshanak Derakhshandeh, Arash Jalali, Mohammad Jahani

**Affiliations:** 1 Hematology, Oncology and Stem Cell Transplantation Research Center, Tehran University of Medical Sciences, Tehran, IR Iran

**Keywords:** Hematopoietic Stem Cell Transplantation, Leukemia, Beta-Thalassemia

## Abstract

**Background:**

Hematopoietic stem cell transplantation (HSCT) is a new window to therapy of many diseases. From March 1991 through April 2011, a total of 3237 HSCT were performed in the Hematology-Oncology and Stem Cell Transplantation Research Center, affiliated to Tehran University of Medical Sciences. Here we report 20 years experience of HSCT.

**Objectives:**

Our strategy and aim include the protraction of cytogenetic and molecular biological diagnostic tests, the expansion of the first Iranian Cord Blood Bank (ICBB) and development of the first Iranian Stem Cell Donor Program (ISCDP), and improvement the researches in new therapeutic fields.

**Patients and Methods:**

Totally, 3237 patients were undergone HSCT. Of these transplants, 2205 were allogeneic stem cell transplantation, 1016 autologous and 16 syngeneic. Among 2205 patients who were undergone allogenic-HSCT, 34 received cord blood stem cells as stem cell source for transplantation. It is important to point out that cord blood bank at our center provides reliable storage of cord blood stem cells for our patients. Stem cell transplantation was performed for treatment of various diseases such as acute myelogenous leukemia, acute lymphoblastic leukemia, chronic myelogenous leukemia, chronic lymphoblastic leukemia, beta-thalassemia major, sickle- cell thalassemia, sickle- cell disease, multiple myeloma, myelodysplasia, mucopolysaccharidosis, paroxysmal nocturnal hemoglobinuria, non-Hodgkin’s lymphoma, Hodgkin’s disease, severe aplastic anemia, plasma cell leukemia, Niemann-Pick disease, Fanconi anemia, severe combined immunodeficiency, congenital neutropenia, leukocyte adhesion deficiencies, Chediak-Higashi syndrome, osteopetrosis, histiocytosis X, Hurler syndrome, amyloidosis, systemic sclerosis, breast cancer, Ewing's sarcoma, testicular cancer, germ cell tumors, neuroblastoma, medulloblastoma, renal cell carcinoma, nasopharyngeal carcinoma, ovarian cancer, Wilms’ tumor, rhabdomyosarcoma, pancreatoblastoma, and multiple sclerosis. Also, we had 220 cellular therapies for post-myocardial infarction, multiple sclerosis, cirrhosis, head of femur necrosis, Diabetes Mellitus and GvHD treatment. 45 patients were undergone retransplantation in this center.

**Results:**

About 78.2% of the patients (2530 of 3237) remained alive between one to 211 months after stem cell transplantation. Nearly, 21.8% (707) of our patients died after stem cell transplantation. The main causes of death were relapse, infection, hemorrhagic cystitis, graft-versus- host disease and etc.

**Conclusions:**

In Iran, HSCT has been successfully adapted in routine clinical care. Recently, new methods such as double cord blood and haploidentical transplantation have been used to treat many life-threatening diseases.

## 1. Background

The term “Hematopoietic stem cell transplantation” has completely replaced recently with Bone Marrow Transplantation, because of using new sources of stem cells other than bone marrow alone (1). By increase of our knowledge about source and development process of stem cells (2), we can use easier ways like extracting stem cells from peripheral blood instead of bone marrow aspiration and biopsy which is an invasive method (3). Nowadays, peripheral blood as a feasible and easily available source of stem cell is used widely. We can extract stem cells by apheresis the peripheral blood of donor. On the other hand, cord blood cells as another rich source of premature stem cells has rapidly growing use in many parts of the world. It has been considered to solve the legal, ethical and social issues (4,5). Hematopoietic stem cell transplantation (HSCT) is considered as the best treatment option for several malignant, non-malignant hematologic and genetic diseases with the aim of achieving a cure or prolonging survival (6-8). The stem cells also can be used in the prevention and treatment of radiation injuries (9). Over the last two decades, HSCT has seen rapid expansion in use and a constant evolution in its technology (10). Despite the side effects and consequences of HSCT, like drug toxicities, graft- versus- host disease (GvHD) and complications of immunosuppressive drugs, it is still the only cure option for many hematologic or solid organ malignancies.

Iran has a population estimated more than 75 million people (11). The incidence of transplantable disease is estimated to be two per 100,000 or 1400 patients per year. The cost of transplantation procedures is borne by insurance companies and private and some charity sources. Hematology-Oncology and Stem Cell Transplantation Research Center is affiliated to Tehran University of Medical Sciences located in Shariati Hospital, Tehran, Iran. In 1991,our center started its activity. During this time,this center helped patients who need chemotherapy and stem cell transplantation. In parallel, the center augmented new data gathering and data management system. It is in association with other significant transplantation centers of the world to reach new aspects of therapeutic methods and protocols. Using the results of beneficial clinical trials can also be helpful in managing diseases which transplantation is still experimental in their therapy. Our center communicates with the International Blood and Marrow Transplantation Registry (IBMTR) and European group of Blood and Marrow Transplantation (EBMT) members. In association with these organizations, our center has been gathering databases of patients who have undergone transplantation and cooperates with the other centers in specific scientific and research fields (10).Also,the center is a member of the Asian Pacific Blood and Marrow Transplantation (APBMT).

## 2. Objectives

Our strategy and aim include the protraction of cytogenetic and molecular biological diagnostic tests, the expansion of the first Iranian Cord Blood Bank (ICBB) and development of the first Iranian Stem Cell Donor Program (ISCDP), and improvement the researches in new therapeutic fields.

## 3. Patients and Methods

On March 3, 1991,the first hematopoietic stem cell transplantation was performed in this center. From March 1991 through April 2011, a total of 3237 first hematopoietic stem cell transplantations were carried out.

### 3.1. Patients

The male/female ratio of patients was 1331/874 in allogeneic, 613/403 in autologous, and 11/5 in syngeneic group patients. The median age of patients was 20 years (range: 0-63) in allogeneic, 33 years (range: 2-71) in autologous, and 20 years (range: 11- 36) in syngeneic.During that time, 2205 patients were treated by myeloablative and non-myeloablative chemotherapy regimen followed by allogeneic stem cell transplantation (SCT), at the Hematology-Oncology and Stem Cell Transplantation Research Center.

The transplanted diseases which are sorted by frequency include: acute myelogenous leukemia (n=590), thalassemia major (n=508), acute lymphoblastic leukemia (n=244), chronic myelogenous leukemia (n=240), severe aplastic anemia (n=183), Fanconi anemia (n=62), myelodysplastic/Myeloproliferative syndromes/ paroxysmal nocturnal hemoglobinuria (PNH) (n=57), non-hodgkin’s lymphoma (n=31), multiple myeloma (n=13), leukocyte adhesion deficiencies (n=12), osteopetrosis (n=11), chronic lymphoblastic leukemia (n=8), Hodgkin’s disease (HD) (n=6), severe combine immunedeficiency (n=5), sickle cell thalassemia (n=4), Chediak-Higashi syndrome (n=3), breast cancer (n=3), Niemann-Pick disease (n=3), Hurler syndrome (n=3), renal cell carcinoma (n=2), sickle- cell disease (n=2), neuroblastoma (n=1), plasma cell leukemia (n=1), Histiocytosis- X (n=1), rhabdomyosarcoma (n=1) and congenital neutropenia (n=1).

### 3.2. Donors

The donor types of SCTs were Human–leukocyte- antigen (HLA) histocompatible siblings (n=2054), HLA-matched other relatives (n=73), HLA-mismatched siblings (n=56), and HLA mismatched unrelated (n=22). The patients who were treated by allogeneic SCT received a median of 9.44×108/kg nucleated cells infused (range: 0.93 – 33.29×108/kg).

From March 1991 through April 2011, 1016 patients were treated by intensive chemotherapy followed by reinfusion of non-cryopreserved autologous stem cells.Thepatients had the following diseases: multiple myeloma (n=280), AML (n=228), HD (n=215), NHL (n=182), ALL (n=25), neuroblastoma (n=16), Ewing's sarcoma (n=9), breast cancer (n=8), plasmacell disorders (n=8), testicular cancer (n=7), amyloidosis (n=7), germ cell tumor (n=6), systemic sclerosis (n=4), medulloblastoma (n=4), ovarian cancer (n=3), plasma cell leukemia (n=2), nasopharyngeal carcinoma (n=1), Wilms' tumor (n=1), pancreatoblastoma (n=1), and multiple sclerosis (n=1) (Table 1).

**Table 1:** Diseases Transplanted With Graft Types

	Graft Type			Total
	Allogeneic	Autologous	Syngeneic	
**AML^a^**	590	228	5	823
**Thalassemia Major**	508			508
**ALL^a^**	426	25	8	459
**Lymphomas**	37	397	1	435
**Multiple Myeloma**	13	280		293
**CML^a^**	240			240
**Severe Aplastic Anemia**	189		2	191
**Inherited Abnormalities of RBC**	73			73
**Solid Tumors**	7	62		69
**MDS^a^/MPS^a^**	51			51
**Disorders of Immune System**	33			33
**Plasma Cell Disorders**	2	19		21
**Inherited Disorder of Metabolism**	21			21
**Other Leukemia**	11			11
**Auto-Immune Diseases**		5		5
**Histiocytic Disorders**	4			4
**Total**	2205	1016	16	3237

^a^Abbriviations: AML, acute myelogenous leukemia; ALL, acute lymphocytic leukemia; CML, chronic myelogenous leukemia; MDS, myelodysplastic syndrom ; MPS , mononuclear phagocyte system

### 3.3. Infused Cell Counts

The patients who were undergoneautologous SCT received a median of 6.96×108/kg (0. 1 – 33.25×108/kg) nucleated marrow cells. During this period, 16 patients were treated by syngeneic SCT. Their diseases were ALL (n=8), AML (n=5), severe aplastic anemia (n=2), and NHL (n=1). They received a median of 6.26×108/kg (3.8– 9.22×108/kg) nucleated marrow cells infusion. Stem cells were obtained and then infused without purging.

### 3.4. Post-transplant Care

Patients were nourished by special and sterilized food and special diet according to each patient characteristics and needs. They were observed closely for GvHD complications and their treatment. All patients were treated in completely isolated rooms during the pre and post-transplantation period. They were conventional, private and High-Efficiency Particulate- Arresting (HEPA) filtered rooms with minimal entertainment to avoid patient depression during hospitalization period (12).

### 3.5. Data Gathering

The Hematology - Oncology and Stem Cell Transplantation Research Center has a data management office, consisting of gathering data group which collect data from the stem cell transplantation wards, check reports for completeness and correctness and identify missing data.They will report missing fields and after completing them, enter data report forms into the computer database program (software version 3.2.4 which meets the needs of hematopoietic stem cell transplantation centers) for statistical analysis. Preparation of statistical reports and writing articles are the other line of works. This office controls the quality of data-entry process. It is responsible for advising patients with providing information about stem cell transplantation process and its consequences and its needed personal cares.

Endpoints: In this study, endpoints were disease-free survival (DFS), overall survival (8), morphologic leukemia relapse (hematologic and/or extramedullary), acute and chronic GvHD. OS was measured as the time interval between the date of transplantation and the date of death due to any cause; surviving patients were censored at the date of last contact. DFS was defined as time to clinical or hematologic relapse or death from any causes other than relapse; patients who remained alive in complete remission were censored at time of last contact. For analyses of acute GvHD (aGvHD), patients alive at100thday without having experienced aGvHD considered censored. Chronic GvHD is defined only for patients surviving at least 100 days after transplantation; patients without chronic GvHD (cGvHD) were censored at last contact.

Definitions: the total hematopoietic stem cell transplantation number, here, points to the number of first-time patients treated with hematopoietic stem cell transplantation. After first HSCT, if relapse or rejection occurs, in special cases, we use unplanned HSCT known as re-transplantation. Donor lymphocyte infusion (DLI) is a method of treatment for patients with recurrent or persistent malignancy after allogeneic HSCT, with an infusion of additional lymphocytes obtained from the original donor, without the cover of immunosuppressive agents (13).Cell therapy is a technology that introduces the new cells (potential stem cells) to adiseased or malfunction organ in order to replace the new healthy and functional tissue with damaged one.

### 3.6. Statistical Analysis

The groups were compared by using Mann-Whitney U test for continuous variables and chi-square test for categorical variables. Overall and disease-free survival curves were calculated by the Kaplan-Meier method (14); and groups were compared by using the Log-Rank test statistic (15). Death before 100^th^day considered as a competing event for acute GvHD. Death after 100^th^ day was considered as a competing event for chronic GvHD. Groups were compared by the Grays' method in the competing risk settings (16).The level of significance was set to 0.05. The packages cmprsk (17) and survival (18) in the R software (18) were used to conduct the statistical analyses.

## 4. Results

**Figure 1.**
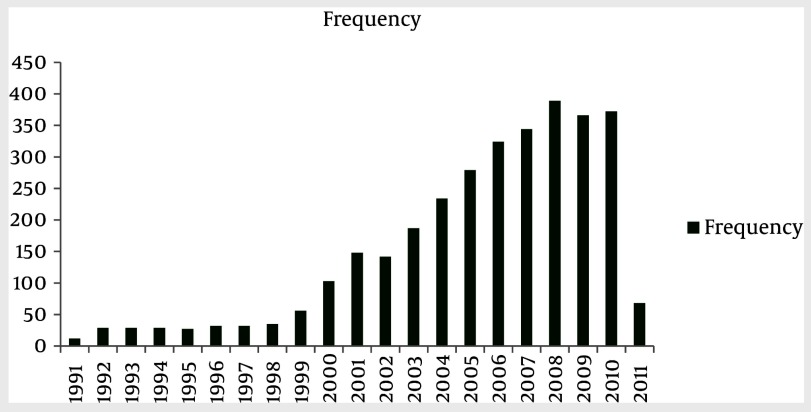
Total Number of Patients Transplanted Each Year

**Figure 2:**
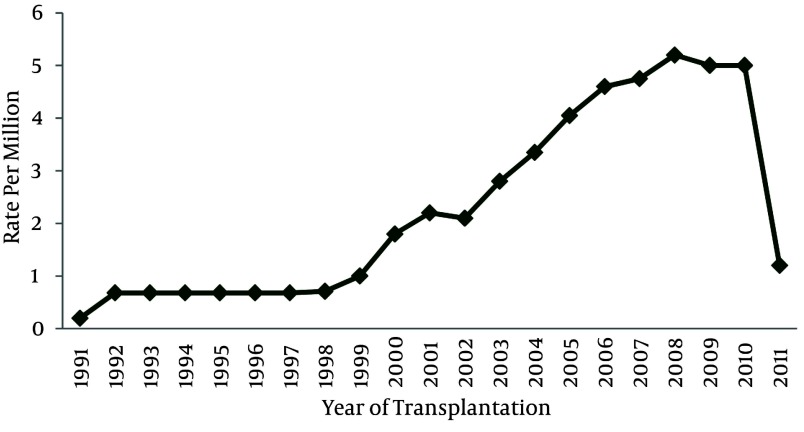
Rate of Transplantation Was Done During 20 Years

**Figure 3:**
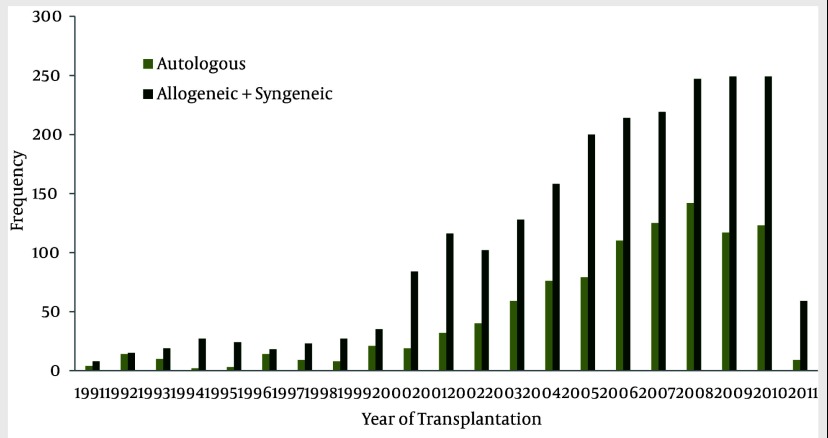
Number of Autologous and Allogeneic HSCT Done Per Year

**Figure 4:**
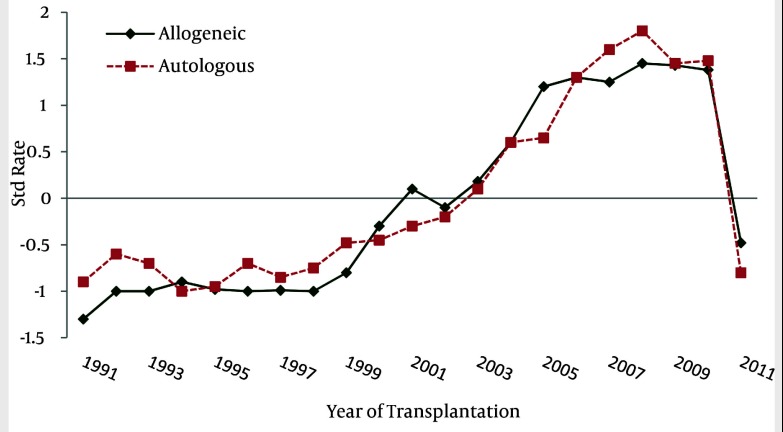
Rate of Autologous and Allogeneic HSCT Per Year

HSCT has increased about ten-fold compared to the last decade. The hematopoietic stem cell transplantation rate during the past 20 years was 49.4 transplants per one million inhabitants. Population data has been obtained from the Statistical Center of Iran (11). Also, HSCT rates have increased from 1991 to 2011 (Figure 1 and 2). The small number of transplanted patients in 2011, was due to reportingthe results of the study within the first 4 months of the year, only. Of the 3237 first transplant patients, 2205 (68.1%) received allogeneic and 1016 (31.4%) autologous HSCT (Figure 3). There has been continuous increment in HSCT activity for both allogeneic and autologous during this period of time, but the standard rate of transplantation has stayed the same in both allogeneic and autologous (Figure 4).

**Figure 5:**
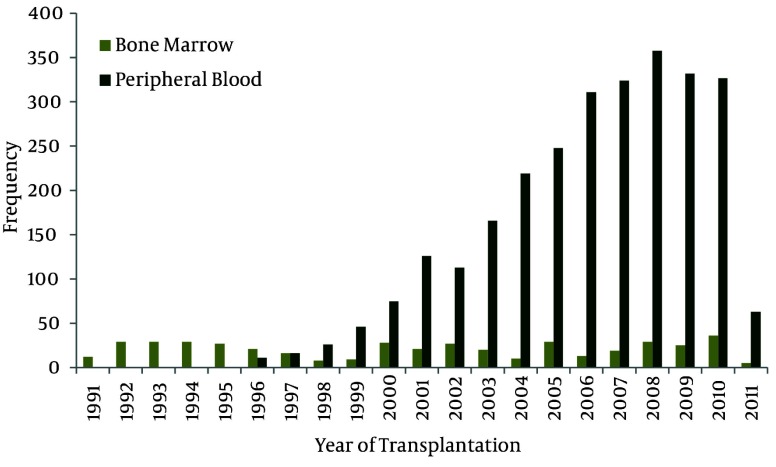
Sources of HSCT Done Each Year

Of the 2205 allogeneic first HSCT patients, 1783 (80.9%) received peripheral blood, 375 (17%) bone marrow, 13 (0.6%) combined peripheral blood and bone marrow, 34 (1.5%) cord blood and 87 (4%) mesenchymal with bone marrow or peripheral blood as stem cell providing source. Of the 1016 autologous patients with their first HSCT, 946 (93.1%) received peripheral blood, 65 (6.4%) bone marrow and 5 (0.5%) mixed bone marrow and peripheral blood as stem cell sources (Figure 5). Since the first cord blood transplantation was done at 1998, 34 HSCT was done from cord blood source in this center. The greatest number of cord blood transplantation occurred during 2009 and 2010. All of the cord blood HSCTs were allogeneic and there was no autologous cord blood HSCT. We organized the first public cord blood bank of Iranian population in order to use this rich and free source of stem cells for patients’ treatment.

### 4.1. Allogeneic HSCT

We had 2205 patients who underwent an allogeneic HSCT. The most common disease was acute myeloid leukemia (n=590, 26.8 %), followed by thalassemia (n=508, 23%) and then acute lymphoblastic leukemia (n=426, 19.3%) (19).

### 4.2. AML

**Figure 6:**
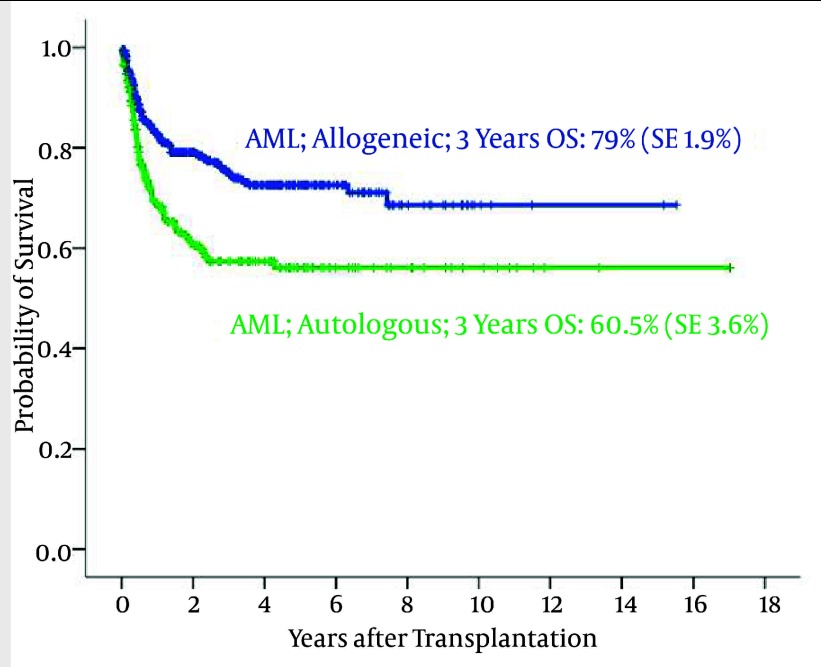
The Probability of Overall Survival in AML Patients According to Graft Type

590 AML patients (277 women and 313 men) underwent allogeneic HSCT. The median age at transplantation was 27 years (range 1–59). Overall, 437 patients were in first CR, 96 in second CR and 23 in third or higher CR. Other recipients were in primary induction failure (PIF) or relapse at the time of transplantation. 462 (78.3%) patients are still alive. The 3-year Overall Survival (8) and Disease- Free Survival (DFS) were 79% (SE: 1.9%) and 71.9% (SE: 2%), respectively. Acute GvHD occurred in 317 patients (53.7%), including grade I (108), grade II (123), grade III (72) and grade IV (14). The DFS and OS in AML patients with allogeneic were significantly more than the patients with autologous SCT (P < 0.0001) (Figure 6).

### 4.3. Thalassemia

**Figure 7:**
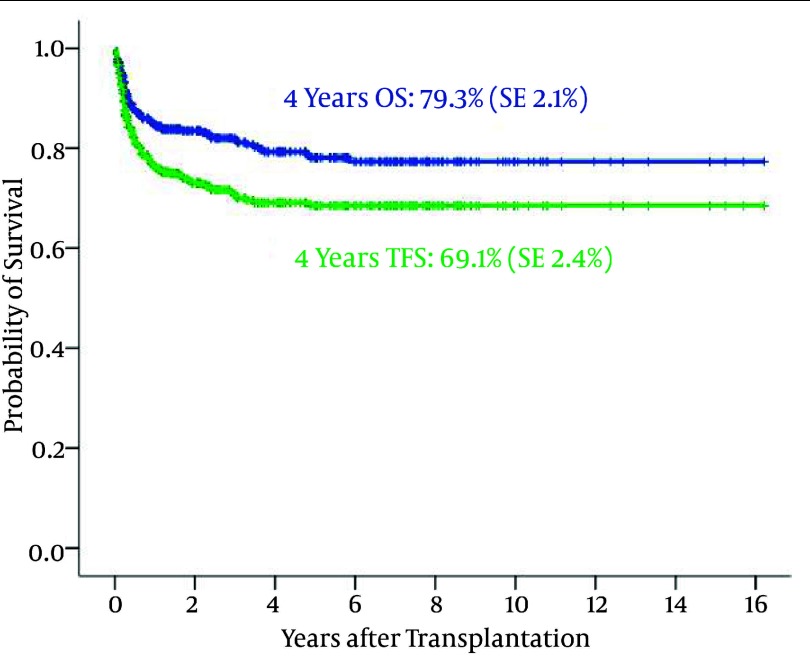
Overall Survival and Thalassemia Free Survival in Transplanted Beta- Thalassemia Major Patients

508 (217 female and 291 male) with Beta-thalassemia major underwent allogeneic HSCT. The median age at transplantation was 7 years (range 2–29). The 4-year OS and DFS are 79.3% (SE: 2.1%) and 69.1% (SE: 2.4%), respectively (Figure 7). 316 patients developed acute GvHD, which was grade I (n= 88), grade II (n= 118), grade III (n= 82) and grade IV (n= 28).

### 4.4. ALL

**Figure 8:**
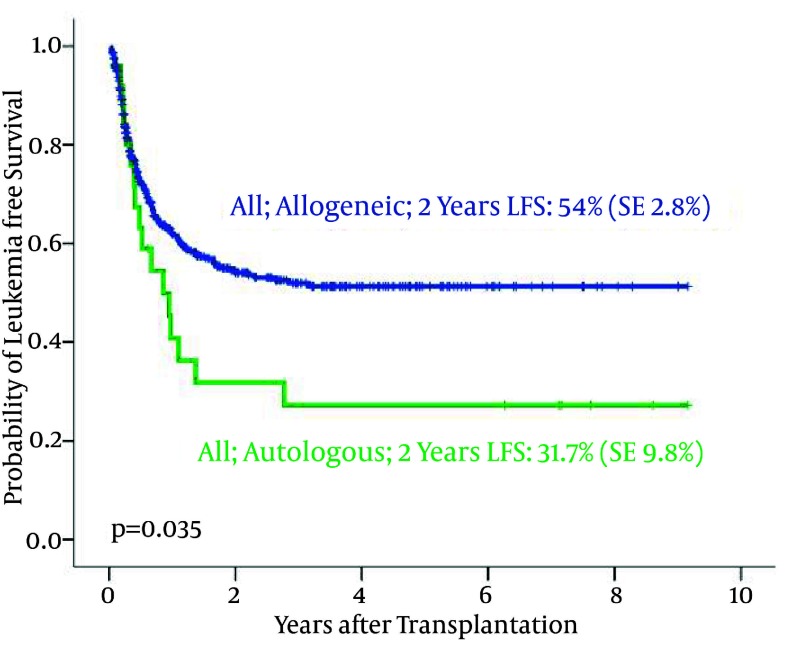
Probability of Leukemia- Free Survival in Transplanted ALL Patients According to Graft Type

426 ALL patients (138 female and 288 male) underwent allogeneic HSCT. The median age at transplantation was 20 years (range 2–52). 2-year OS and DFS were 63.4% (SE 2.7%) and 54% (SE 2.8%), respectively. 257 patients developed aGVHD which was grade I (82), grade II (108), grade III (49) and grade IV (18). Same as AML, the DFS and OS in ALL patients with allogeneic were significantly more than the patients with autologous SCT (Figure 8).

### 4.5. Auto-HSCT

We had 1016 patients who underwent an auto-HSCT. The underlying disease was multiple myloma in 280 cases, acute myeloid leukemia in 228, HD in 215, NHL in 182, and other diseases in 111 cases (19).

### 4.6. Multiple Myloma

**Figure 9:**
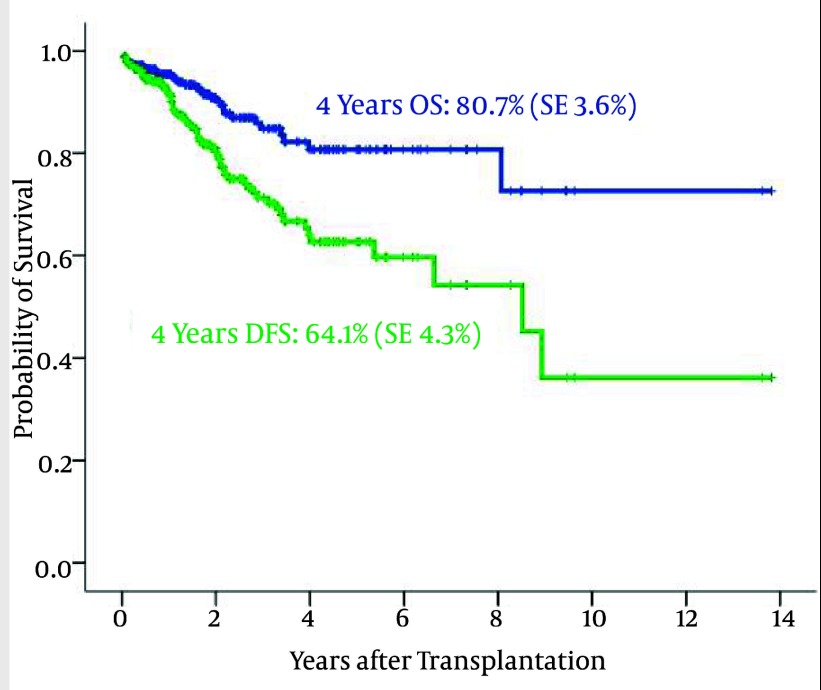
The 4-Year Overall Survival and Disease- Free Survival in Transplanted Multiple Myeloma Patients

Totally, 280 patients with multiple myeloma (MM) underwent autologous HSCT (107 female and 173 male). The median age was 50 years (range 20–71). 84 patients underwent autologous HSCT in outpatient ward. Disease status at HSCT was as follows: 87.1% were in CR, 11.4% had PR and others (1.5%) were in progression or relapse. The 4-year OS and PFS were 80.7% (SE3.6%) and 64.1 % (SE4.3%), respectively (Figure 9).

### 4.7. Hodgkin’s Disease

**Figure 10:**
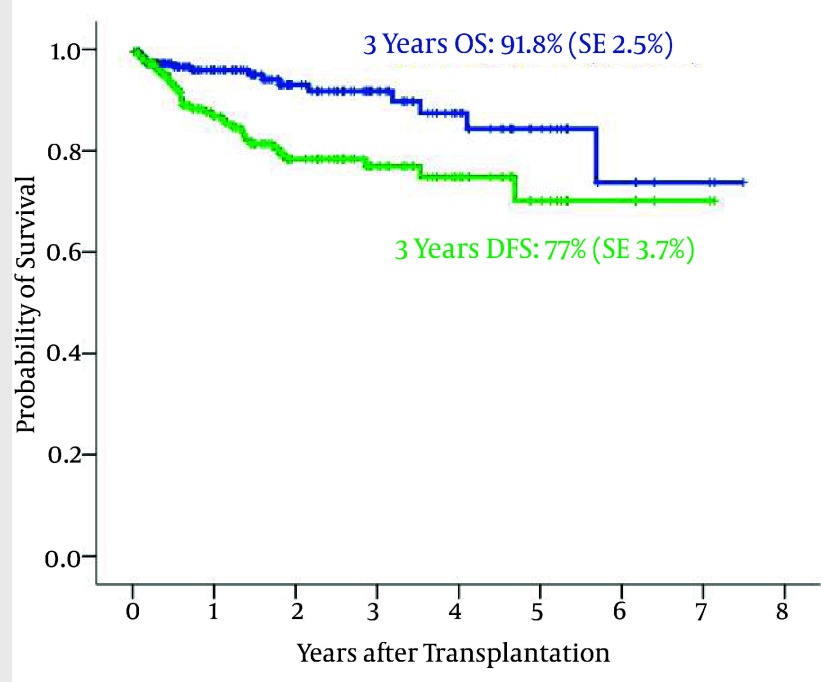
The 3-Year Overall Survival and Disease- Free Survival in Transplanted Hodgkin's Disease Patients

215 patients (83 female and 132 male) underwent autologous HSCT for HL. Nineteen patients underwent autologous HSCT in outpatient ward. It is a new and encouraging methodwhich isused for special patients who have good condition in home care and low distance from hospital. It results in less nosocomial infections and faster recovery period. The median age was 25 years (range 12 – 60). The 3-year OS and DFS were 91.8% (SE 2.5%) and 77% (SE 3.7%), respectively (Figure 10). We observed15 patients died during follow up. The main cause of death was relapse in 10 patients. The other causes of death were cardiac toxicity and infection.

### 4.8. AML

228 patients underwent an auto-HSCT for AML. The median age was 24 years (range2-68). At time of transplantation, 194 patients were in CR1, 20 in CR2, 2 in third or higher CR, 8 with primary induction failure and 4 in relapse. 3-year DFS and OS were 51.2% (SE 3.5%) and 60.5 (SE 3.6%), respectively.

## 5. Discussion

Hematopoietic-SCT represents a potentially curative treatment modality in a range of hematologic malignancies (20). According to statistical reviewing during the past 20 years in Hematology-Oncology and Stem Cell Transplantation Research Center, hematopoietic stem cell transplantation numbers and rates have increased, especially after the year 2000. It's important to note that the small number of transplant patients in 2011, is due to reporting the results of the study up to the fourth month of the year, only. It is remarkable thatthenumber of allogeneic HSCT aremore than autologous HSCT, but both of them have increased with a constant ratio. It can be concluded that the number of HSCT for acute myelogenous leukemia and acute lymphoblastic leukemia was more frequent amongst all disorders; also, the major type of HSCT in these diseases was allogeneic, which is similar to the survey of the European Group for Blood and Marrow Transplantation (EBMT) (21). Peripheral blood was the major source of hematopoietic stem cell transplantation which wasthe same as in the EBMT survey.

The most common donor type in allogeneic transplantations was full human- leukocyte antigen (HLA) -matched siblings. We found that allogeneic SCT is an effective and useful treatment for patients with malignant, non-malignant, and genetic diseases and as well as solid tumor (22). Allogeneic HSCT with the graft-versus-leukemia effect is a reasonable method with good results in AML treatment (23). Allogeneic hematopoietic stem cell transplantation (HSCT) has been reported to be a successful curative treatment for patients with AML (24, 25). Newer trials go toward use of allogeneic HSCT in special risk groups of AML to reduce the relapse rate and increase survival (26). The DFS and OS in our AML patients underwent autologous HSCT was similar to Martins C. et al study (27)Autologous HSCT is effective in standard risk AML patients without HLA- matched donor and rises the survival. Allogenic HSCT with a humanleukocytes antigen (HLA) matched sibling donor or unrelated matched donor should be considered in patients with unfavorable risk AML and select patients with intermediate risk AML as consolidation treatment in first CR. Allogeneic HSCT substantially reduces the risk of relapse and provides a potentially curative treatment.The patients with favorable characteristics are not offered allogeneic HSCT after first CR, as this has not improved OS or disease-free survival (24,28).

Allogeneic HSCT is the only cure for major beta-thalassemia patients (29,30).Even some studies suggest unrelated allogeneic HSCT for high risk thalassemia patients who had not matched sibling donor (31). Also, we found that our thalassemic patients have an OS of 79.3% (SE=2.1%) and DFS of 69.1% (SE=2.4%) at 4 years after SCT. The DFS of thalassemia in our center is close to the result of thalassemia in Italy, however we conclude the OS and DFS in all Pesaro classes of thalassemia classification (32). Use of HSCT in treatment of thalassemia results in reducing health burden costs on patient and health system of society, especially in developing countries. In addition to thalassemia, HSCT has been used in treatment of refractory and metastatic solid tumors with theory of graft- versus – solid tumor effect (33).We also used of HSCT in treatment of some metastatic solid tumors like neuroblastoma, breast cancer, Ewing sarcoma and others. Some of these cases has acceptable survival with tolerable GvHD effects.Developing more available sources to find suitable donors, like cord blood bank and stem cell donor bank, conclude in more donors available. More donors available means more lives will save. We try to extend our organization to provide better and more facilities with lower costs for patients who needed the expensive cancer therapies.
